# HIV-1 is budded from CD4+ T lymphocytes independently of exosomes

**DOI:** 10.1186/1743-422X-7-234

**Published:** 2010-09-16

**Authors:** In-Woo Park, Johnny J He

**Affiliations:** 1 Department of Microbiology and Immunology, Indiana University School of Medicine, Indianapolis, IN 46202, USA; 2 Center for AIDS Research, Indiana University School of Medicine, Indianapolis, IN 46202, USA

## Abstract

The convergence of HIV-1 budding and exosome biogenesis at late endosomal compartments called multivesicular bodies has fueled the debate on whether HIV-1 is budded from its target cells and transmitted in the form of exosomes. The point of contention appears to primarily derive from the types of target cells in question and lack of a well-defined protocol to separate exosomes from HIV-1. In this study, we adapted and established a simplified protocol to define the relationship between HIV-1 production and exosome biogenesis. Importantly, we took advantage of the newly established protocol to unequivocally show that HIV-1 was produced from CD4+ T lymphocytes Jurkat cells independently of exosomes. Thus, this study not only presents a simplified way to obtain highly purified HIV-1 virions for identification of host proteins packaged into virions, but also provides a technical platform that can be employed to define the relationship between exosome biogenesis and budding of HIV-1 or other viruses and its contributions to viral pathogenesis.

## Text

Exosomes were initially identified as small membrane vesicles from immature red blood cells [1] and have since been detected in various mammalian cells, tissues and physiological fluids [see a recent review [2]]. They are originated from multivesicular bodies through direct fusion with plasma membrane [3,4], with sizes ranging between 30 and 100 nm [5,6]. Several important functions have been attributed to these small vesicles, these include protein homeostasis [7], humoral immune response [5,8-10], cell-cell interaction [11,12], and anti-tumor activity [6]. In addition, exosomes have also been proposed to play an important role in HIV-1 budding and infection [13], as exosomes and HIV-1 converge at the endosomes and share similar host lipid and protein compositions [10,14]. In macrophages and dendritic cells, HIV-1 was shown to bud into the endosomes [15-20] and secreted in the form of exosomes [21-23]. Recently, a consensus has emerged that HIV-1 does not bud into endosomes but to an external compartment [24,25]. To the contrary, the findings in CD4+ T lymphocytes are quite inconsistent and uncertain. Some studies suggest that HIV-1 is budded from T cell plasma membrane and does not involve endosomes and exosomes [26-31], while others show that T cells produce HIV-1 in close association with exosomes, similarly to that in macrophages and dendritic cells [32-34]. The inconsistency concerning the relationship between HIV-1 budding and exosome biogenesis conceivably is likely due to cross-contamination of each other during isolation and purification as a result of their indistinguishable sizes and densities [35,36]. Thus, to define the precise role of exosomes in HIV-1 budding, transmission and other virol-immunological processes, it is imperative to establish a simplified and reproducible protocol that allows clear separation of exosomes from HIV-1.

Several ways have been exploited to study HIV-1 interaction with exosomes. The general approach is a step-wise protocol, which is composed of first brief low-speed centrifugation to remove cells and cell debris from the cell culture supernatant, then filtration by passing the cleared through a 0.22 nm filter, and lastly high-speed centrifugation to obtain exosomes and/or HIV-1 virions. The presence of exosomes, HIV-1, or both is evaluated by detection of exosome markers, and HIV-1 viral antigens, and electron microscopic imaging. In this study, we introduced a modified protocol that allows successful separation of HIV-1 virions from exosomes. Similar protocols have been widely employed to isolate or concentration HIV-1 virions.

**Figure 1:**
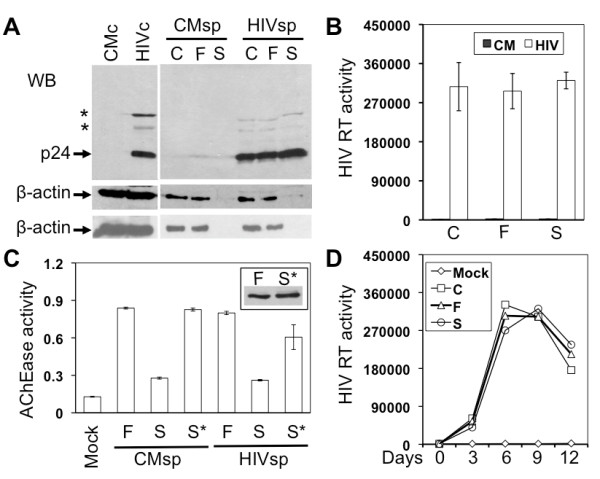
**HIV-1 production and exosome biogenesis in Jurkat cells**. **A**. Jurkat cells were infected with HIV-1 HXB2 viruses (HIV) or mock infected (CM). Cells (c) were harvested and culture supernatants (sp) were collected 9 days after infection. Culture supernatants were first cleared of cells and debris by low-speed centrifugation, followed by filtration and further 20% sucrose sedimentation. The virion preparations from these three steps were C, F, and S, respectively. Cell lysates and virion preparations were subjected to Western blotting using antibodies against HIV-1 p24 or β-actin (upper: sucrose banding for 1 hr; lower: sucrose banding for 2.5 hr). *: p24 precursors. **B**. HIV-1 RT assay of three virion preparations. **C**. Acetylcholinesterase (AChe) activity assay of the virus preparations F and S as well as the sucrose cushion from sucrose sedimentation (S*). **D**. Jurkat cells were inoculated with each of three virus preparations (corresponding to 10,000 cpm of RT activity) and monitored for virus infection and replication.

Briefly, Jurkat cells were infected with HIV-1 HXB2 viruses equivalent to 10,000 cpm reverse transcriptase (RT) activity and cultured for 7-9 days when virus replication was peaked (data not shown). The cell culture supernatant was collected and first centrifuged at 800 *g *for 10 min to remove cells and cell debris. The cleared supernatant was then passed through a 0.22 μm filter (Corning, NY) to ensure complete removal of smaller cell debris. The pass-through supernatant was loaded onto 1 ml 20% sucrose in PBS and centrifuged with a SW55Ti (Beckman, NY) at 238,000 *g *for 90 min to obtain the virion preparation (S). To compare virion compositions, a same volume of the cleared supernatant from the first centrifugation and the pass-through from the filtration was loaded onto 1 ml PBS and subjected to the same last step high-speed centrifugation to obtain virion preparation C and F, respectively. All three virion preparations were suspended in the SDS-PAGE sample buffer for Western blot analysis. Using the highly abundant β-actin protein as an exosomal marker [2,37,38], we detected exosomes in virion preparations C and F, but not in virion preparation S (Figure 1A). Importantly, we detected a comparable level of HIV-1 p24 in all three virion preparations (Figure 1A), as well as a comparable level of RT activity among all three virion preparations (Figure 1B). These results together show that the high-speed centrifugation with the 20% sucrose cushion at the last step gives rise to HIV-1 virions completely free of exosomes, refuting the HIV-1 Trojan exosome hypothesis. We also included the lysates from HIV-1-infected Jurkat cells (HIVc) and mock-infected Jurkat cells (CMc), as well as the pellets of supernatants from mock-infected Jurkat cells (CMsp), as controls in the experiments. Longer high-speed centrifugation at the last step, i.e., 2.5 hr, did not change the β-actin distribution pattern (bottom, Figure 1A).

**Figure 2:**
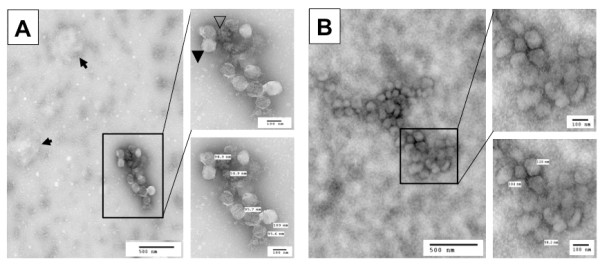
**EM micrographs**. **A**. The virus preparation (F) was fixed, diluted 10-fold and negative stained for EM imaging. Open arrowhead: exosomes; closed arrowhead: HIV-1 virions; arrows: membrane vesicles. **B**. The virus preparation S. Both images in **A **and **B **were representative of multiple EM images.

To confirm that the new protocol did lead to successful separation of HIV-1 virions from exosomes, we further analyzed virion preparations F and S for the presence of exosomes using the other well-documented exosome marker, acetylcholinesterase (AChe) [1,31]. We found a significant level of AChe activity in virion preparation F but a much lower level of AChe activity in virion preparation S (Figure 1C). The Ache activity in preparation F and S showed little changes between the mock- and HIV-1-infected samples. To ensure that exosomes were completely separated from HIV-1 virions and thereby remained in the sucrose cushion (S*), we further analyzed the AChe activity in the sucrose cushion and detected a level of AChe activity in the sucrose cushion comparable to that in preparation F (Figure 1C), verifying a clear separation of HIV-1 from exosomes by the new protocol. This was further supported by Western blotting analysis that β-actin was detected in preparation S* with a comparable intensity to that in preparation F in mock-infected samples, indicating that almost all exosomes in preparation F were separated from virions and recovered in preparation S* (Insert in Figure 1C). We obtained similar results from HIV-infected samples (data not shown). Using another exosome marker, heat shock protein 70 (Hsp70) [38-41], we also obtained similar results (data not shown). To further ascertain independent release of HIV-1 virions from exosomes, we fixed and negative stained both F and S virion preparations and visualized them using transmission electron microscopy. Preparation F contained particles of at least three different sizes: 80-120 nm HIV-1 virions (closed arrowhead), 30-100 nm irregularly shaped exosomes (open arrowhead), and larger other membrane vesicles (arrow) (Figure 2A), with about 83.7 ± 4.3% exosomes and 15.8 ± 3.2% HIV-1 virions from a total of eight randomly selected EM fields in multiple EM images. In comparison, preparation S had 80 - 120 nm HIV-1 virions free of any sizes of membrane vesicles (Figure 2B), with 4.3 ± 3.2% exosomes and 93.5 ± 5.7% HIV-1 virions. Furthermore, we determined whether there were any differences in the infectivity of these three virion preparations. To this end, we infected Jurkat cells with each of the viruses of the same amount of RT activity and monitored virus infection and replication in these cells. There were little differences of viral replication kinetics among these three virion preparations (Figure 1D). Thus, unlike the findings from dendritic cells that exosomes-associated HIV-1 virions are more infectious [21], these results indicate that the presence of exosomes does not affect the HIV-1 infectivity in Jurkat cells.

In summary, all these experiments show that HIV-1 virions obtained from the new protocol are free of exosomes and provide conclusive evidence that HIV-1 budding and exosome secretion in Jurkat cells are independent from each other. Of note are two other published protocols that have also been shown to produce exosomes-free HIV-1 virions. One involves use of iodixanol gradient sedimentation followed by fractionation [31]. Besides its requirement of the special agent iodixanol, the fractionation manipulation in this protocol is quite laborious. The other protocol is to use CD45 magnetic beads to deplete CD45-containing exosomes from HIV-1 virion preparations [28]. This protocol is clearly not applicable to analysis of exosomes and HIV-1 virions produced from cells that express little or no CD45. Thus, this study not only presents a simplified way to obtain highly purified HIV-1 virions free of exosomes or other cellular vesicles for basic HIV-1 virological studies, but also provides a technical platform that can be employed to further define the relationship between HIV-1 budding and exosome biogenesis in other HIV-1 target cells such as macrophages and dendritic cells and its contributions to HIV-1 pathogenesis.

## Competing interests

The authors declare that they have no competing interests.

## Authors' contributions

IWP designed, performed experiments and prepared the manuscript; JJH designed and prepared the manuscript. Both authors read and approved the final version of the manuscript.

## Authors' information

**In-Woo Park**, Ph.D., Assistant Research Professor, Center for AIDS Research and Department of Microbiology and Immunology Indiana University School of Medicine, Indianapolis, IN 46202, USA

**Johnny J. He**, Ph.D., Professor and Director, Center for AIDS Research and Department of Microbiology and Immunology Indiana University School of Medicine, Indianapolis, IN 46202, USA
